# Knowledge and Use of Evidence-Based Practice in Psychology in the Clinical Practice of Brazilian Psychologists: A Cross-Sectional Study

**DOI:** 10.3390/healthcare13040431

**Published:** 2025-02-17

**Authors:** Tamara Melnik, Jorge Sinval, Vanessa Dordron de Pinho, José Antônio Spencer Hartmann Junior, Margareth da Silva Oliveira, Fernanda Machado Lopes

**Affiliations:** 1 Department of Evidence-Based Health, Paulista School of Medicine, Federal University of São Paulo, São Paulo 04023-062, SP, Brazil; tamelnm@gmail.com; 2 Business Research Unit (BRU-IUL), Instituto Universitário de Lisboa (ISCTE-IUL), 1649-026 Lisbon, Portugal; 3 Faculty of Philosophy, Sciences and Languages of Ribeirão Preto, University of São Paulo, Ribeirão Preto 14040-901, SP, Brazil; 4 National Institute of Education, Nanyang Technological University, 1 Nanyang Walk, Singapore 637616, Singapore; 5 Faculdade de Medicina, Universidade de Lisboa, 1649-028 Lisbon, Portugal; 6 Department of Psychology, State University of Rio de Janeiro, Rio de Janeiro 20550-900, RJ, Brazil; vanessanessapsi@gmail.com; 7 Faculty of Medicine, University of Pernambuco, Recife 50670-901, PE, Brazil; jose_spencer@yahoo.com.br; 8 Department of Psychology, Graduate Program in Psychology, Pontifical Catholic University of Rio Grande do Sul, Porto Alegre 90619-900, RS, Brazil; margathremel@gmail.com; 9 Department of Psychology, Graduate Program in Psychology, Federal University of Santa Catarina, Florianópolis 88040-970, SC, Brazil; femlopes23@gmail.com

**Keywords:** evidence-based psychology, evidence-based practice in psychology, clinical practice, Brazil, clinical psychology

## Abstract

**Background/Objectives**: The use of scientific evidence for the diagnosis and treatment of mental disorders is crucial for achieving optimal clinical outcomes and providing high-quality care. This study investigates the knowledge, sources of evidence, and attitudes of Brazilian clinical psychologists regarding evidence-based practice in psychology (EBPP) and discusses barriers to its implementation. **Methods**: A total of 696 Brazilian clinical psychologists participated in an online questionnaire designed to assess their understanding of EBPP, their familiarity with scientific platforms/databases, and their professional development investments. Latent class analysis (LCA) was conducted, which can be used to identify subgroups of psychologists with similar patterns of professional and training characteristics. **Results**: The results indicate that while psychologists recognized the importance of EBPP for effective patient care, there was a significant gap in understanding its fundamental principles and concepts. Many participants reported the limited use of scientific databases, missing opportunities to access the latest research advancements. A lower percentage of psychologists consistently implemented EBPP in their clinical practice, highlighting a gap between knowledge and application. Four latent classes emerged from the LCA: I—experienced/established professional psychologist; II—academic psychologist; III—supervised traditional psychologist; and IV—young professional psychologist. **Conclusions**: This study emphasizes the need for better integration of EBPP into psychology curricula and continuing education programs. Enhancing clinical psychologists’ understanding and proficiency in EBPP can promote evidence-based decision-making and improve the quality of mental health care in Brazil. Efforts should be made to familiarize psychologists with reliable scientific databases, equip them with skills to critically appraise research, and foster a culture of lifelong learning and professional development. Additionally, it is essential to develop strategies tailored to the distinct profiles of professionals identified in this study, considering their training sources, reference usage, and knowledge of EBPP.

## 1. Introduction

Evidence-based practice in psychology (EBPP) requires psychologists to access, appraise, and integrate the research literature with clinical experience and clients’ values, contexts, and preferences [1], promoting an effective psychological practice that enhances public health [2]. Continuing education in the latest scientific evidence is essential to mitigate biases and prevent overestimation of treatment effects in psychotherapy [3]. There is a consensus in the literature about the gap between the amount of research evidence in psychology and the use of this evidence by clinical psychologists [4]. Muran and Lipner [5] highlight that understanding changing processes in psychotherapy, including common factors and transdiagnostic approaches, can significantly enhance the application of evidence-based principles in clinical practice.

In 2015, the American Psychological Association’s Division 12 updated the criteria for rigorous and reliable evidence in psychology. This update included systematic reviews as the primary level of evidence. The National Institute for Health and Care Excellence (NICE) has also established a process in which experts, including clinicians, researchers, and consumers, evaluate scientific evidence to determine the best treatment options for physical and mental disorders. The treatments deemed as frontline interventions for each condition are expected to be provided to patients in England by the National Health Service. NICE also monitors the extent to which the National Health Service complies with these recommendations. NICE has found that Evidence-Based Psychological Treatments (EBPT) are effective frontline interventions for a wide range of mental disorders, either alone or combined with other treatments. Despite this, a significant proportion of adult patients in the UK do not receive evidence-based mental healthcare, with estimates ranging from 10 to 40% [6,7,8]. In this context, Knapp et al. [9] emphasize the importance of aligning therapeutic interventions with research-derived knowledge to fulfill ethical obligations and promote patient well-being.

The head of the National Institutes of Mental Health in the United States noted that, despite less funding, psychosocial interventions may offer more promising outcomes than pharmacological treatments [10]. Some international initiatives establishing evidence-based psychological treatments for most mental disorders have been challenging [7,11], as, unfortunately, there is still a lot of resistance by clinical psychologists toward using the scientific literature to support their practice [12]. Critics suggest that EBPP risks over-standardizing psychology, potentially devaluing clinical intuition. Furthermore, some mention that the time spent reading and understanding the scientific literature is too long, in addition to the investment in learning interventions with which they are unfamiliar [4,12]. Still, there is a discussion about reliable evidence and implementing research data in reality.

Factors influencing the effective implementation of evidence-based psychology remain poorly understood. Melnik and Atallah [11] suggest that the implementation of evidence-based psychology involves a complex interaction of factors and characteristics across various levels, including social, organizational, economic, and political dimensions, as well as individual levels related to both therapists (e.g., their theoretical orientations) and patients. Considering the high costs to the health system and the great demand for mental health services, the evaluation of interventions has been a concern on the political agendas of several countries. In a study conducted in Uganda, East Africa, key barriers identified among lecturers not applying evidence-based practice (EBP) included resistance to change, lack of resources, inadequate organizational support, and insufficient locally generated research. To enhance EBP adoption, the study suggests incorporating EBPP training into healthcare curricula, developing robust “train-the-trainers” programs, and ensuring the availability of necessary resources [13]. Similarly, a scoping review on EBP implementation in healthcare in China found that limited knowledge, insufficient skills and resources, and incomplete procedures or pathways were identified as the main obstacles to EBP implementation. On the other hand, the most frequently cited facilitator was leadership support. Education and training emerged as the key strategies to promote EBP adoption among healthcare professionals and patients [14].

In Brazil, Law No. 12.401 [15] and Law No. 10.216 [16] both provide “legal support for the use of the best scientific evidence in public healthcare as the basis for diagnosing and treating diseases in this country” [17] (p. 624). EBPP aligns with the legislative and ethical principles of maximizing benefit and responsible caring when selecting interventions with evidence of efficacy and effectiveness [3], yet it has not become widely adopted by healthcare professionals, including psychologists. However, little is known about the extent to which Brazilian psychologists are aware of and utilize EBPP, as well as their specific educational needs in this regard.

Melnik and Atallah [11] have shown that factors such as the learning process associated with the implementation of evidence-based guidelines, the healthcare providers’ attitudes and beliefs, leadership support and integration of recommendations at an academic level, resource constraints, collaboration, and established networks all affect the implementation process of EBPP. Knowledge translation is a dynamic and interactive process that includes synthesis, dissemination, exchange, and ethically sound application of knowledge to improve health [18]. Thus, since EBPP involves knowledge translation, as well as innovation and disengagement, requiring changes in paradigms and attitudes, its incorporation is understandably gradual.

### Purpose of the Current Study

The theoretical framework chosen to underpin this research consists of the Scientist-Practitioner Model and the Knowledge Translation Framework, as these paradigms emphasize the integration of scientific evidence with clinical expertise and client values. Considering the complexities and the recognized importance of EBPP, this study aims to explore Brazilian clinical psychologists’ knowledge, of EBPP, their sources of training, and its application in practice. Specifically, it seeks to identify different profiles of clinical psychologists by examining their sources of training investment, the references they consult to support their clinical practice, their use of databases, and their knowledge of EBPP. The research questions guiding this study are as follows: (1) Do different profiles of clinical psychologists exist based on their sources of training investment, references sought, use of databases, and knowledge of EBPP? (2) If such profiles exist, how are these groups characterized in terms of their therapeutic approaches, client demographics, therapy modalities, length of treatment, and the types of disorders they treat?

## 2. Methods

### 2.1. Sampling

A cross-sectional correlational research survey design was used to meet the study objectives. A non-probability convenience sample was obtained from the target population: Brazilian psychologists working in clinical psychology and registered with the Federal Council of Psychology. The email addresses were obtained from the OrientaPsi online platform. Additional email addresses were obtained through a snowball approach, where psychologists recommended colleagues who met the inclusion criteria.

### 2.2. Measures

A structured questionnaire composed of two parts was used for data collection. The first part aimed to gather the demographic data of the participants: gender, age, academic level, university attended, and any previous EBP training received. The second part contained open- and closed-ended questions investigating psychologists’ understanding of evidence-based practice in psychology, their use of use of scientific databases, familiarity with research tools, and their forms of investment in specialization or professional development. The questionnaires were presented in the Portuguese language. It is estimated that each respondent would need between five to ten minutes to complete the questionnaire.

### 2.3. Procedure

All procedures performed in studies involving human participants followed the institutional research committee’s ethical standards and the 1964 Helsinki Declaration and its later amendments or comparable ethical standards. This study was submitted to and approved (20 December 2019) by the university ethics committee (n. 3.786.486; CAAE: 26776019.3.0000.5336), and informed consent was obtained from all participants. After receiving information about the research, individuals were directed to the questionnaire only after confirming that they had read and understood the research procedures described in the informed consent form and that they were aware their data would always be handled anonymously and confidentially. The research was disseminated through professional and social networks on online platforms, primarily in psychology-related groups on Facebook. Additionally, it was directly shared via email with psychologists holding active professional registrations with a Regional Council of Psychology (CRP).

An invitation letter containing the online access link to the questionnaire was sent, and it remained available from January 2020 to July 2021 via the Qualtrics platform [19]. When accessing the link, participants were directed to the Informed Consent Form, which they needed to read and accept before proceeding to the questionnaire. The professional registration number of the Regional Council of Psychology was requested to ensure that data were being collected exclusively from qualified professionals in psychology.

### 2.4. Data Analysis

For the open-ended responses, a thematic analysis was conducted to identify and organize barriers and perceptions related to EBPP among Brazilian clinical psychologists. A coding framework was developed and reviewed by multiple researchers, categorizing responses into themes such as “access to databases”, “training deficiencies”, and “cultural resistance”.

Descriptive statistics were performed to describe the participants’ demographic, professional, and training characteristics. A latent class analysis (LCA) was conducted to identify subgroups of psychologists sharing similar professional and training profiles [20]. LCA is based on the assumption that there is an underlying and unobserved categorical variable that organizes a population into mutually exclusive groups [21]. The *poLCA* package [22] was used to conduct the LCA. Two parsimony measures (that account for model complexity) were used to assess model fit: the Akaike Information Criterion (AIC) [23] and the Bayesian Information Criterion (BIC) [24]. The lower the value, the better the fit of the model to the data. Due to its simplicity, BIC is usually more appropriate for regular LCA models [25,26]. The χ^2^ goodness of fit and likelihood ratio chi-square (*G*^2^) statistics were also used to evaluate the fit of model to the data [27]. These statistics are based on the principle of observed versus expected cell counts. For AIC, BIC, χ^2^, and *G*^2^, lower values indicate a better statistical fit. All statistical analyses were conducted in the statistical programming language *R* [28] via the integrated development environment, *RStudio* [29].

## 3. Results

### 3.1. Sample Characteristics

A total of 696 clinical psychologists registered with the Federal Council of Psychology responded to the survey. Most of the sample of psychologists consisted of women (73.3%) who were Brazilian (99.7%), living in the Southeast (45.5%) and Southern (26.8%) regions of Brazil, with a mean age of 40 years and and average of 12 years since graduation. Concerning clinical practice, most preferred seeing adults (94.3%) in the individual therapy modality (99.6%), lasting from six months to two years (70%) and primarily using cognitive behavioral approaches (37.5%) or psychoanalysis (26.9%). Participants could select more than one response for these items. The mean time spent working in the clinical area was 11 years. The most common pathologies cited as more frequent among the clients seen by the responding psychologists were anxiety disorders (91.2%) and mood disorders (73.4%).

### 3.2. Investment in the Improvement and Access to Evidence-Based Practice

Regarding investment in training and improvement, most responded that they invest in books (89.9%), take training or specialization courses (78.3%), or study on their own (76.7%). Notably, less than half responded that they invest in supervision (48.3%). Regarding the type of reference that they seek to support their care, the majority reported consulting books (91.2%) or scientific articles (81.2%). Finally, regarding evidence-based practice, 66.8% responded that they had already heard of it, but 51.1% claimed to using this practice to support their care. In turn, 59.5% of psychologists responded that they usually use some database as a reference source for their clinical practice. Table 1 outlines participants’ investment in training and EBPP access.

Among those who responded that they had heard about EBP from other sources, most cited scientific articles (*n* = 31), followed by training or extension courses (*n* = 24), colleagues (*n* = 18), congresses or events (*n* = 17), and internet websites (*n* = 10). Many other sources have been cited (e.g., SECAD program, NGOs, podcasts, institutional affiliation, “I do not remember”) at low frequency.

The analysis of the open-ended question “What do you understand by EBPs?” indicated that many participants have some notion about these practices. Still, only a minority understood what it means to act using scientific evidence. Briefly, only 19 participants in this study seemed to know the tripod characterizing EBP, according to the following reports: “Professional performance that considers scientific knowledge, professional expertise, and client preferences in clinical decision making”; “Union between scientific evidence, therapist expertise, and patient particularities to choose the best treatment for the patient”. Most of the sample only articulated that EBPP refers to using practices with scientific evidence of efficacy/effectiveness (*n* = 322), for example: “A practice whose effectiveness and efficacy were tested through scientific experiments and positive evidence for its use was observed. That is, this practice has proven scientific evidence that it works”; “Use the best available scientific knowledge to guide their clinical decisions”. The other responses suggest a lack of understanding of EBP.

A preliminary analysis of the other open-ended question of this study was conducted, which questioned participants about the obstacles regarding EBPs in psychology. Out of the 616 responses obtained for this question, 109 indicated that they did not know the subject and did not provide an opinion. This indicates that many Brazilian clinical psychologists recognize that they do not know what EBPs are. The scenario of the lack of dissemination of EBPs in the Brazilian context is better elucidated in other responses. Many participants attributed the obstacles to the training of psychologists, as reflected in the response, “not well-established in Brazil, starting with the course syllabus of the undergraduate programs themselves”. Others mentioned that not all psychological approaches value scientific evidence, as in the following excerpts: “The epistemological basis of the approach adopted by the professional” and “The lack of concern of professionals of most approaches regarding the validation of the therapeutic strategies used”. Several responses reported the lack of dissemination of the topic, the difficulty accessing scientific articles, and databases that are not easy to use, as follows: “Access to data in Portuguese, greater scientific dissemination, support from regional councils”. Also, several participants mentioned the lack of support, interest, and funding for research in Brazil, which was identified in statements such as “Lack of incentive and research funding” and “Low budget for research development in Brazil”. Taken together, these data indicate that the little knowledge and low adherence to EBP in Brazil by Brazilian clinical psychologists may be associated with the lack of dissemination of this proposal.

### 3.3. Latent Class Analysis (LCA)

Four models were tested from two to five latent classes using 21 variables of interest, as no theoretical background existed to determine the number of classes. The model with four classes was the one that presented the best fit in terms of BIC, while the model with five latent classes presented lower AIC, G^2^, and χ^2^. The four-classes model was selected for its superior balance between interpretability and fit. The two-classes model oversimplified the diverse professional behaviors and educational investments of psychologists, failing to capture the nuanced differences among them. The three-classes model, while slightly more detailed, still lacked sufficient granularity to meaningfully distinguish between groups, resulting in overlapping characteristics. Conversely, the five-classes model became overly complex, making it difficult to derive clear interpretations and actionable insights from the class distinctions. However, in terms of interpretability, the four-classes solution seems more plausible (Table 2). The four-classes model effectively balanced complexity and clarity, allowing for distinct profiles based on the types of learning and references used by psychologists, such as reliance on books, supervision, and evidence-based practices. As such, the four-latent-classes solution was adopted and discussed (the complete materials on the latent class analysis can be accessed upon request by the corresponding author).

The relative frequencies (in proportion) for each of the 21 categorical variables are shown in Table 3.

Table 4 provides a detailed characterization of the joint sample and the proposed EBPP latent classes.

The first class consisted of *experienced/established professional psychologists*. This class contained professionals with more experience (*M* = 14.20 years since graduating) who primarily rely on books, self-studying, and courses/specializations. Most do not use databases or have not heard about evidence-based practices. They used, as references, books, clinical experience, and scientific articles. Among these professionals, supervision is not used as a reference.

The second latent class consisted of *academic psychologists*. These professionals are younger (*M* = 39.41 years), with an average of 13.66 years since graduating; they invest more in books, self-studying, congresses, short-term courses, and postgraduate education. They do use databases and have heard about evidence-based practices. They use, as references, books, scientific articles, and evidence-based practices.

*Supervised traditional psychologists* constituted the third latent class. These professionals have, on average, 13.47 years since graduating and invest more in books, courses/specializations, and supervision. They are less likely to use databases and are unfamiliar with the term EBPP. They use, as references, books, clinical experience, and supervision. They do not look for EBPP as a source for their sessions.

Finally, the fourth latent class was composed of *young professional psychologists.* This class comprises younger (*M* = 37.77 years) and less experienced professionals (*M* = 10.95 years since graduating) who invest more in books, self-studying, congresses, short-term courses, courses/specializations, and supervision. They do use databases and are familiar with the term EBPP.

Across the four latent classes, there is a notable predominance of female psychologists, with Class I (*experienced/established professional psychologists*) being 72% female, Class II (academic psychologists) also being 72%, Class III (*supervised traditional psychologists*) being 72%, and Class IV (*young professional psychologists*) being 77%. In terms of state of residency, Class I (*experienced/established professional psychologists*) has the highest frequency in São Paulo at 33%, while Class II (*academic psychologists*) shows a slightly lower frequency at 21%. Class III (*supervised traditional psychologists*) has a relevant concentration in São Paulo at 46%, and Class IV (*young professional psychologists*) has 30% residing in the state.

The four classes exhibit distinct differences in therapeutic approaches, client demographics, therapy modalities, and types of disorders treated. *Experienced/established professional psychologists* predominantly employ cognitive behavioral therapy (36%) and psychoanalysis (26%), indicating a reliance on well-established therapeutic frameworks. They primarily work with adults (96%) and have a high frequency of clients with anxiety disorders (92%). *Academic psychologists* show a preference for cognitive behavioral therapy (57%) and primarily engage with adult clients (89%). These psychologists face a similar frequency of clients with anxiety disorders (87%). The group *supervised traditional psychologists* has 93% of them treating adults and focusing on a range of disorders, with anxiety being the most frequent (93%). The last, latent class, *young professional psychologists*, combines various approaches, with cognitive behavioral therapy (32%) and psychoanalysis (33%) being notable. They work mainly with adult clients (95%) and focus more frequently on anxiety disorders (94%) and mood disorders (76%).

## 4. Discussion

This study aimed to investigate the level of knowledge and utilization of EBPP by Brazilian clinical psychologists, revealing that, although most are aware of EBPP, fewer have received training, and even fewer implement it frequently in practice. This is likely due to limited access to research databases and training programs, hindering their ability to stay current and change clinical practices. Furthermore, this study reveals that a low percentage of psychologists in Brazil has used clinical research studies to change their clinical practices.

Atzil-Slonim [30] notes that recent advances in understanding the therapeutic process underscore the significance of both interpersonal dynamics and individual client factors in promoting successful outcomes, which are critical for effective EBPP implementation. The integration of research evidence, clinical judgment, and client preferences is a core component of evidence-based practice [2], yet it is often perceived as emphasizing research evidence to the exclusion of practitioner expertise and client choice [31]. This perception contributes to a research-practice gap that is evident in various psychological disciplines, including clinical psychology and neuropsychology [32,33]. Our findings support this notion, as many Brazilian psychologists report imbalances between these elements, often prioritizing clinical judgment and client preferences due to the practical demands of their work environment.

Research methods that support practitioner-researchers in applied practice contexts, such as problem-based methodology, may help bridge this gap by systematically enhancing practice through active engagement with an individual’s decision-making processes [31]. On the other hand, in this study, participants were asked where they found out about EBPP, and 28.9% had never heard about it before. More than one-third of participating students in our study cited that they had not received previous training on EBPP, even though they all had successfully passed the psychologist research course offered at their universities. There is inconsistency in how research in psychology courses is taught, often omitting EBPP practice. Thus, tertiary institutions offering psychology degrees in Brazil must critically review their current educational strategies related to EBPP and improve them to enhance students’ understanding and implementation of EBP in clinical settings.

Similar findings were discussed by McHugh and Barlow [34]. The article provides a comprehensive review of the state of dissemination and implementation of evidence-based psychological treatments. The authors explore the challenges associated with promoting the adoption and use of such treatments in clinical practice and discuss various strategies for improving the dissemination of evidence-based practices. They conclude that while significant progress has been made in recent years, there is still much work to be done to ensure that all patients have access to the most effective and evidence-based treatments available. A study conducted in Canada evaluated 26 types of psychotherapies and found that about 50% of them had not been evaluated in terms of efficacy and effectiveness [35]. The study recommends that psychological associations increase their focus on EBPP and provide more resources to support its implementation in clinical practice.

The implementation of EBPP can be a complex process, but some fundamental strategies to promote EBPP include (1) building a culture of EBPP training programs, (2) providing training and support in psychology undergraduate degrees, and (3) engaging stakeholders in mental health. Despite the recognized importance of evidence-based practice, considerable variability remains among practitioners. There is a notable disparity in understanding and applying EBPP among practicing psychologists, with many relying on traditional sources such as books and clinical experience rather than the scientific literature [32,36].

Creative and enjoyable strategies are fundamental to encouraging students’ commitment to and learning about EBPP. One of these effective strategies is teaching the EBPP process. There is a consensus that implementation typically involves the following steps: identifying the problem; conducting a literature search; evaluating the evidence; applying the evidence; monitoring progress; and documenting the process [37].

Implementing EBPP involves identifying the issue, conducting a literature search, evaluating the evidence, applying it, and monitoring progress. After conducting the literature search, the next step is to critically evaluate the quality of the evidence found by some resources. Tools like the Cochrane Risk-of-Bias Tool (RoB 2) [38], Newcastle–Ottawa Scale (NOS) [39], QUADAS-2 [40], and the ROBINS-I [41] can help to assess the quality of evidence. These tools are used by researchers, systematic reviewers, and others to evaluate the quality of clinical trials and to assess the risk of bias that may impact the validity of the results. The choice of tool will depend on the type of study being evaluated, and it is important to use the appropriate tool for the specific study design. This may involve considering factors such as the study’s design, sample size, and potential sources of bias.

The evidence gathered in the literature search should then be applied to the specific case at hand. This may involve considering the client’s individual characteristics and making a clinical judgment informed by the research evidence and clinical expertise. After implementing EBP, monitoring the client’s progress and making any necessary adjustments to the treatment plan is crucial. This may involve re-evaluating the evidence and considering other factors, such as the client’s response to the treatment. Finally, it is important to document the process of implementing EBPP, including the search for evidence, evaluating the evidence, and applying the evidence to the specific case. This can help ensure that the process is transparent and can be easily replicated in future cases.

The four proposed classes illuminate the reality of EBPP in Brazil. The existing diversity in training investment and sources for patient sessions clearly indicate the presence of different viewpoints. The groups of *experienced/established professional psychologists* and *supervised traditional psychologists*—less oriented towards EBPP—are composed of professionals who seem to rely more on clinical experience and traditional sources of information. Conversely, the groups of *academic psychologists* and *young professional psychologists* seem more familiar with EBPP. Psychology practice, teaching, and research in Brazil are evolving [42]. Professional experience appears to play a role; freshly graduated practitioners should be more familiar with databases, more sensitive to the importance of an evidence-based approach, and consequently have a better understanding of the scientific research [43]. This is corroborated by our results; however, even among younger professionals, two groups emerge: one more aligned with the scientist-practitioner model (i.e., *academic psychologists*), and another more interested in the clinical science model (i.e., *young professional psychologists*). In line with this, Luebbe et al. [36] found that attitudes towards EBPP appear to be associated with the professional model students aim to follow, with those planning primarily clinical research careers exhibiting a more favorable view towards EBPP compared to those planning primarily clinical practice careers.

Our analysis reveals that *academic psychologists* are more closely aligned with evidence-based practices, whereas *experienced/established psychologists* often prioritize traditional sources and clinical experience. This understanding offers a foundation for designing targeted interventions tailored to specific professional profiles. The *experienced/established psychologists* limited engagement with databases, and unfamiliarity with EBPP may hinder their ability to integrate contemporary practices into their work. To address this, training programs could emphasize the importance of EBPP and provide resources that bridge the gap between traditional practices and modern evidence-based approaches. Workshops that highlight successful case studies of EBPP in practice may also encourage this group to adopt new methods. Regarding the *academic psychologists* group, they are likely to benefit from advanced training that focuses on the application of EBPP in various settings. Encouraging their participation in research initiatives and collaborative projects can enhance their skills and confidence in implementing EBPP, which may ultimately lead to more innovative therapeutic approaches. The reliance of *supervised traditional psychologists* on supervision suggests a potential avenue for integrating EBPP into their practice through enhanced supervisory relationships. Training programs could focus on integrating EBPP discussions into supervision sessions, allowing for these psychologists to explore how evidence-based practices can complement their existing frameworks. Additionally, providing resources that clarify the relevance and application of EBPP within their supervisory contexts can help overcome barriers to adoption. The familiarity of *young professional psychologists* with databases and EBPP suggests that they are more adaptable to integrating evidence-based practices. However, they may still require support in dealing with the complexities of implementing these practices in real-world settings. Training programs could focus on practical applications of EBPP, offering hands-on workshops that address specific challenges faced in clinical settings.

Older psychologists, represented in the groups *experienced/established professional psychologists* and *supervised traditional psychologists*, may be less prone to adopting EBPP due to their reliance on traditional methods and skepticism about the efficacy of newer approaches. To facilitate a smoother transition, it is essential to understand their concerns and provide tailored training that respects their expertise while introducing evidence-based concepts. In contrast, younger and less experienced psychologists in the groups *academic psychologists* and *young professional psychologists* tend to have more positive attitudes toward EBPP, influenced by contemporary training methods. However, their inexperience can hinder the application of these practices in clinical settings. Implementing mentorship programs that connect them with established professionals open to learning about EBPP could enhance their confidence and skills.

In the international context, the United Kingdom exemplifies the integration of NICE guidelines and systematic clinician training as cornerstones of public healthcare practices [44]. In the United States, the APA has promoted EBPP through initiatives such as continuing education and certification programs [1]. In Canada, challenges in psychotherapy evaluation have led psychological associations to take steps to enhance EBPP dissemination [3]. These insights provide a broader context for our findings and serve as a foundation for proposing strategies adapted to Brazil’s unique socio-cultural and systemic characteristics.

The adoption of EBPP may encounter several obstacles. The main barriers identified in our study, based on the reports of clinical psychologists, include structural, cultural, and institutional challenges, such as limited research funding, inadequate integration of EBP into psychology curricula, resistance to change among experienced professionals, restricted access to scientific literature, personal skepticism about EBPP’s value, and insufficient training in its practical application [32,33]. Some psychologists might be less familiar with electronic datasets and/or have limited access to research.

To improve access to scientific databases for psychologists in Brazil, specific initiatives should be explored, such as providing free access to more databases/platforms by national governments or professional organizations. The Cochrane Database of Systematic Reviews—currently freely available for all residents of Brazil—illustrates this potential; research indicates that free access significantly boosts demand for review downloads and summary views among healthcare professionals. For many OECD countries, this access could cost less than USD 2 per additional download, leading to substantial savings on existing subscriptions [45]. The Cochrane Database of Systematic Reviews is also freely available in other countries under the HINARI program established by the World Health Organization to enable developing countries to access collections of the biomedical and health literature [46]. Adding Brazil to programs that follow the spirit of the HINARI program can facilitate the access and adoption of evidence-based practices among psychologists. Translating plain-language summaries into local languages can further enhance accessibility and dissemination [45]. Implementing such initiatives can facilitate the adoption of evidence-based practices among psychologists in Brazil.

Barends et al. [47] found similar barriers among 2,789 management practitioners in Belgium regarding the use of EBP. In terms of training, psychologists may not have received sufficient training in EBPP during their education and training, which can make it difficult for them to apply it in their practice. Horntvedt et al. [48] found that interactive and clinically integrated teaching strategies, such as case studies and simulations, effectively promote EBPP knowledge and skills in nursing education. Supervision seems to be an important in developing evidence-based practice competencies during psychology training programs, but empirical investigations into this area are limited [49].

Personal and cultural factors may also play an important role, as psychologists’ personal beliefs may conflict with EBPP (e.g., they may believe their clinical experience and intuition are sufficient). Another possible justification is that the therapeutic approach they were trained in might be less oriented towards evidence-based practices. Individual factors, such as openness to change, may also play a role, which, together with the complexity of integrating new evidence into the current practice, can make it challenging to shift from traditional approaches to evidence-based ones [47].

This study represents the first attempt to identify evidence-based profiles among clinical psychologists in Brazil. A primary limitation is the use of a non-probability sampling method, such as convenience and snowball sampling, which may introduce selection bias during recruitment. As a result, the sample may not be representative of the broader population of Brazilian clinical psychologists, particularly given Brazil’s vast cultural diversity [50]. However, it is noteworthy that the distribution of psychologists by state in our sample closely aligns with the relative frequencies reported by each Regional Psychology Council in the 2022 Census of Brazilian Psychology. For instance, 28.1% of the 432,173 registered psychologists were in São Paulo [51], which is comparable to the 31% observed in the current study sample. Moreover, while it is likely that all psychologists have internet access and possess devices (e.g., smartphones or computers) to complete the survey, it is not expected that all of them share the same level of familiarity with participation in online surveys. This aspect may influence their willingness to participate in such studies and, consequently, impact the representativeness of the sample.

Future research should collect larger random samples in different settings and from different areas of specialization. It is also recommended to investigate the barriers that prevent psychology students from implementing EBPP, beyond simply not receiving training on it. New studies should link EBPP knowledge with its actual use in clinical practice and analyze the implementation process. This would provide valuable insights into how frequently and effectively EBPs are applied and identify factors that influence their practical application. Conducting qualitative studies might help examine and understand psychologists’ perceptions, as well as provide suggestions to bridge the gap between education and practice. Experimental studies are needed to test the effect of certain interventions on enhancing the implementation of EBPP among psychologists. To guide both researchers and educators in better supporting the integration of EBP into psychology practice in Brazil, future studies could focus on longitudinal research to assess the long-term impact of EBP training. Furthermore, additional studies are necessary to allow for the support of the four identified profiles. The current study sample comes from one area of psychology specialization (i.e., clinical psychology), which is not enough to assume that these profiles will be the same in other areas of specialization. Other profiles can emerge from contextual factors (i.e., areas of specialization, professional organizations) and individual heterogeneity.

Despite these limitations, the use of EBP by Brazilian psychologists is still seen as a valuable approach to clinical decision-making, as it helps to ensure that clients receive treatments supported by the best available evidence. Gaining knowledge about psychologists understanding and their ability to implement EBPP in clinical settings is essential for national and international psychology educators.

This knowledge might help educators evaluate and improve the current strategies for educating undergraduate students about EBPP. Gaines and Goldfried [52] argue that identifying areas of agreement within the field, such as the common factors in psychotherapy, can bridge the research–practice divide and strengthen the understanding of effective therapeutic principles. Academic administrators and teachers should design their courses to apply EBPP concepts by promoting training courses, workshops, and seminars. For example, research courses should focus more on this topic and should include clinical scenarios that involve the application of EBPP. In addition, clinical courses should include assignments to integrate EBPP within their clinical cases. They should also collaborate to facilitate the implementation of EBPP in clinical settings to overcome any barriers. Moreover, Sinval [53] emphasizes the importance of critically assessing, selecting, and developing/adapting psychometric instruments, which are crucial for ensuring that such instruments used in clinical practice align with evidence-based principles.

Evidence-based practice is essential for psychologists worldwide. However, having strong beliefs about EBPP and its benefits does not necessarily mean that psychologists frequently implement it. On the other hand, providing training courses on EBPP is essential in enhancing its implementation. This means that, to advance psychological science and enhance mental health care for the future, it is vital to incorporate EBPP within the psychology curricula. Specific actionable strategies are proposed for integrating EBPP into the curricula and continuing education. These include developing mandatory EBPP-focused modules in undergraduate and graduate psychology programs, promoting supervision focused on evidence-based practices, and creating accessible platforms to facilitate access to scientific databases. Additionally, offering workshops on research design, the critical appraisal of evidence, and effective database usage, as well as promoting supervised clinical practices that emphasize the application of EBPP principles in real-world scenarios, are essential. Strengthening collaboration between academic institutions and professional councils to standardize EBPP training nationwide is also suggested. Regulatory and educational authorities should establish clear policies that actively promote education based on evidence, such as mandatory continuing education in EBPP for license renewal. It is also critical to teach psychology students the value of evidence-based knowledge and how to access it, evaluate it, and apply it correctly when needed. This can be achieved through rigorous cooperation between universities, clinicians, professors, and students to enhance the implementation process. These proposed measures aim to bridge the gap between knowledge and implementation while promoting a culture of evidence-based decision-making.

## Figures and Tables

**Table 1:** Evidence-based practice.

Variables	Sample (*n* = 696)	
	*n*	%
Sources of training investment		
Books	626	89.9
Training or specialization courses	545	78.3
Studying on their own	534	76.7
Takes short courses	501	72.0
Participates in congresses	391	56.2
Supervision	336	48.3
Study groups	267	38.4
Master’s	236	33.9
Doctorate	90	12.9
Other	42	6.0
References sought to support services		
Books	635	91.2
Scientific articles	565	81.2
Clinical experience	538	77.3
Courses	392	56.3
Supervision	364	52.3
Evidence-based practices	356	51.1
Consulting colleagues	310	44.5
Intuition or what they believe that works	71	10.2
Other	10	1.4
Use of databases as a reference source for clinical practice		
Yes	414	59.5
No	282	40.5
Knowledge of EBPP		
Yes	465	66.8
No	231	33.2
Sources of EBPP information		
Never heard of EBPP	201	28.9
Specialization course	193	27.7
Research courses	146	21.0
In the master’s	139	20.0
In the undergraduate course	134	19.3
In the doctorate	52	7.5
Other	191	27.4

Note. EBPP—evidence-based practice in psychology.

**Table 2:** Fit indices for the tested models.

No. of Latent Classes	*AIC*	*BIC*	*G* ^2^	χ^2^	*df*
2	14,892.21	15,087.66	5,874.816	3,021,205	653
3	14,549.13	14,844.58	5,487.735	1,701,618	631
4	14,412.39	14,807.84	5,306.999	1,360,224	609
5	14,345.28	14,840.72	5,195.886	1,311,061	587

**Table 3:** Proportions of training sources, reference use, and EBPP knowledge by latent classes.

Variables	Latent Class			
	I	II	III	IV
Sources of training investment				
Books	0.86	0.89	1.00	0.80
Studying on their own	0.73	0.78	0.91	0.55
Participates in congresses	0.31	0.80	0.80	0.20
Takes short courses	0.65	0.70	0.92	0.50
Training/specialization courses	0.67	0.80	0.90	0.75
Study groups	0.13	0.36	0.66	0.42
Supervision	0.00	0.33	0.96	0.80
Master’s degree	0.20	0.64	0.38	0.04
Doctorate	0.04	0.33	0.12	0.00
Others	0.03	0.08	0.06	0.08
References sought to support services				
Books	0.90	0.91	0.81	0.98
Clinical experience	0.80	0.63	0.77	0.87
Supervision	0.07	0.33	0.89	0.99
Consulting colleagues	0.38	0.39	0.41	0.58
Intuition or what they believe that works	0.12	0.02	0.07	0.17
Scientific articles	0.70	0.98	0.61	0.88
Evidence-based practices	0.36	0.89	0.03	0.59
Courses	0.46	0.50	0.43	0.79
Others	0.00	0.03	0.01	0.02
Databases	0.40	0.97	0.33	0.61
Knowledge of EBPP				
Yes	0.47	0.99	0.30	0.77

Note. I—experienced/established professional psychologists; II—academic psychologists; III—supervised traditional psychologists; IV—young professional psychologists.

**Table 4:** Descriptive statistics of the joint sample and the suggested latent classes.

Variables	Latent Class				Joint Sample
	I	II	III	IV	
	(*n* = 215)	(*n* = 175)	(*n* = 100)	(*n* = 206)	(*N* = 696)
Age (Years)					
*M* (*SD*)	42.71 (±11.44)	39.41 (±11.11)	42.27 (±11.97)	37.77 (±9.51)	40.35 (±11.10)
Missing	0 (0%)	1 (0.6%)	1 (1.0%)	0 (0%)	2 (0.3%)
Sex					
Male	61 (28%)	49 (28%)	28 (28%)	48 (23%)	186 (27%)
Female	154 (72%)	126 (72%)	72 (72%)	158 (77%)	510 (73%)
Nationality					
Brazilian	215 (100%)	174 (99%)	100 (100%)	205 (100%)	694 (100%)
Other	0 (0%)	1 (1%)	0 (0%)	1 (0%)	2 (0%)
State of Residence					
Acre	1 (0%)	1 (1%)	0 (0%)	1 (0%)	3 (0%)
Alagoas	0 (0%)	1 (1%)	0 (0%)	1 (0%)	2 (0%)
Amapá	0 (0%)	3 (2%)	0 (0%)	0 (0%)	3 (0%)
Amazonas	9 (4%)	9 (5%)	0 (0%)	4 (2%)	22 (3%)
Bahia	2 (1%)	2 (1%)	1 (1%)	6 (3%)	11 (2%)
Ceará	10 (5%)	1 (1%)	0 (0%)	11 (5%)	22 (3%)
Distrito Federal	7 (3%)	10 (6%)	2 (2%)	8 (4%)	27 (4%)
Espírito Santo	1 (0%)	3 (2%)	0 (0%)	1 (0%)	5 (1%)
Goiás	8 (4%)	6 (3%)	2 (2%)	7 (3%)	23 (3%)
Maranhão	1 (0%)	0 (0%)	1 (1%)	1 (0%)	3 (0%)
Mato Grosso	1 (0%)	1 (1%)	0 (0%)	1 (0%)	3 (0%)
Mato Grosso do Sul	1 (0%)	2 (1%)	1 (1%)	1 (0%)	5 (1%)
Minas Gerais	21 (10%)	12 (7%)	4 (4%)	21 (10%)	58 (8%)
Pará	5 (2%)	5 (3%)	0 (0%)	1 (0%)	11 (2%)
Paraíba	4 (2%)	1 (1%)	0 (0%)	2 (1%)	7 (1%)
Paraná	5 (2%)	5 (3%)	7 (7%)	9 (4%)	26 (4%)
Pernambuco	6 (3%)	6 (3%)	4 (4%)	5 (2%)	21 (3%)
Piauí	1 (0%)	2 (1%)	1 (1%)	0 (0%)	4 (1%)
Rio de Janeiro	11 (5%)	12 (7%)	2 (2%)	11 (5%)	36 (5%)
Rio Grande do Norte	2 (1%)	3 (2%)	3 (3%)	3 (1%)	11 (2%)
Rio Grande do Sul	27 (13%)	27 (15%)	17 (17%)	32 (16%)	103 (15%)
Rondônia	3 (1%)	2 (1%)	3 (3%)	1 (0%)	9 (1%)
Santa Catarina	14 (7%)	20 (11%)	5 (5%)	17 (8%)	56 (8%)
São Paulo	71 (33%)	37 (21%)	46 (46%)	61 (30%)	215 (31%)
Sergipe	0 (0%)	2 (1%)	0 (0%)	0 (0%)	2 (0%)
Tocantins	1 (0%)	0 (0%)	0 (0%)	1 (0%)	2 (0%)
Missing	3 (1.4%)	2 (1.1%)	1 (1.0%)	0 (0%)	6 (0.9%)
Years since graduating (Years)					
*M* (*SD*)	14.20 (± 10.22)	13.66 (± 10.25)	13.47 (± 9.98)	10.95 (± 7.81)	13.00 (± 9.62)
Missing	2 (0.9%)	0 (0%)	1 (1.0%)	3 (1.5%)	6 (0.9%)
Therapeutic approach					
Cognitive behavioral therapy	78 (36%)	100 (57%)	17 (17%)	66 (32%)	261 (38%)
Existential–humanistic therapy	31 (14%)	13 (7%)	15 (15%)	24 (12%)	83 (12%)
Psychoanalysis	55 (26%)	23 (13%)	41 (41%)	68 (33%)	187 (27%)
Systemic therapy	26 (12%)	16 (9%)	11 (11%)	16 (8%)	69 (10%)
Integration of various therapies	41 (19%)	20 (11%)	10 (10%)	18 (9%)	89 (13%)
Contextual therapy	4 (2%)	21 (12%)	2 (2%)	10 (5%)	37 (5%)
Jungian therapy	9 (4%)	2 (1%)	13 (13%)	13 (6%)	37 (5%)
Gestalt therapy	21 (10%)	9 (5%)	6 (6%)	10 (5%)	46 (7%)
Other	30 (14%)	35 (20%)	14 (14%)	39 (19%)	118 (17%)
Clients					
Children	72 (33%)	54 (31%)	39 (39%)	81 (39%)	246 (35%)
Adolescents	141 (66%)	101 (58%)	69 (69%)	149 (72%)	460 (66%)
Adults	207 (96%)	156 (89%)	98 (98%)	195 (95%)	656 (94%)
Older adults	101 (47%)	61 (35%)	54 (54%)	83 (40%)	299 (43%)
Therapy modality					
Individual	215 (100%)	174 (99%)	99 (99%)	205 (100%)	693 (100%)
Couple	66 (31%)	37 (21%)	32 (32%)	52 (25%)	187 (27%)
Groups	48 (22%)	46 (26%)	20 (20%)	55 (27%)	169 (24%)
Family	43 (20%)	27 (15%)	17 (17%)	42 (20%)	129 (19%)
Mean length of treatment					
Brief (up to 6 months)	96 (45%)	62 (35%)	22 (22%)	64 (31%)	244 (35%)
Intermediate (up to 2 years)	145 (67%)	118 (67%)	70 (70%)	155 (75%)	488 (70%)
Prolonged (more than 2 years)	49 (23%)	48 (27%)	39 (39%)	84 (41%)	220 (32%)
Disorders					
Anxiety disorders	197 (92%)	152 (87%)	93 (93%)	193 (94%)	635 (91%)
Mood disorders	153 (71%)	124 (71%)	78 (78%)	156 (76%)	511 (73%)
Personality disorders	56 (26%)	52 (30%)	35 (35%)	67 (33%)	210 (30%)
Eating disorders and obesity	36 (17%)	34 (19%)	13 (13%)	42 (20%)	125 (18%)
Substance-related and addictive disorders	31 (14%)	26 (15%)	15 (15%)	41 (20%)	113 (16%)
Schizophrenia and other psychotic disorders	24 (11%)	11 (6%)	13 (13%)	26 (13%)	74 (11%)
Neurocognitive or neurodevelopmental disorders	31 (14%)	34 (19%)	14 (14%)	34 (17%)	113 (16%)
Sexual Dysfunctions	22 (10%)	18 (10%)	18 (18%)	32 (16%)	90 (13%)
Other	26 (12%)	29 (17%)	10 (10%)	27 (13%)	92 (13%)

Note. I—Experienced/established professional psychologists; II—Academic psychologists; III—Supervised traditional psychologists; IV—Young professional psychologists.

## Data Availability

The raw data supporting the conclusions of this article will be made available by the authors on request.
